# Evaluation of the Psychometric Properties of PainChek® in UK Aged Care Residents with advanced dementia

**DOI:** 10.1186/s12877-021-02280-0

**Published:** 2021-05-28

**Authors:** Ivana Babicova, Ainslea Cross, Dawn Forman, Jeffery Hughes, Kreshnik Hoti

**Affiliations:** 1 College of Health, Psychology & Social Care, University of Derby, Derby, UK; 2 University of Derby Online Learning, University of Derby, Derby, UK; 3 Curtin Medical School, Curtin University, Perth, Australia; 4 Division of Pharmacy, Faculty of Medicine, University of Prishtina, Prishtina, Kosovo

**Keywords:** PainChek®, dementia, pain, validation, observational pain assessment

## Abstract

**Background:**

The aim of this study was to further validate PainChek®, an electronic pain assessment instrument, with a population living with dementia in a UK care home.

**Method:**

This study utilised a correlational design to evaluate the psychometric properties of PainChek® when compared to the Abbey Pain Scale (APS). Blinded paired pain assessments were completed at rest and immediately post-movement by a researcher and a nurse. A total of 22 participants with a diagnosis of moderate-to-severe dementia and a painful condition were recruited using opportunity sampling.

**Results:**

Overall, 302 paired assessments were collected for 22 participants. Out of these 179 were conducted during rest and 123 were immediately post-movement. The results demonstrated a positive significant correlation between overall PainChek® pain scores and overall APS pain scores (*r* = 0.818, *N* = 302, *p* < .001, one-tailed), satisfactory internal consistency (α = 0.810), moderate single measure intraclass correlation (ICC = 0.680) and substantial inter-rater agreement (κ = 0.719).

**Conclusions:**

PainChek® has demonstrated to be a valid and reliable instrument to assess the presence and severity of pain in people with moderate-to-severe dementia living in aged care.

## Background

Pain in frail older adults with dementia is a major concern [1], with literature consistently reporting poor treatment and management in terms of inappropriate administration of analgesics and incorrect recognition of presence and severity of pain [2, 3]. Up to 80 % of individuals with dementia living in care homes experience acute or chronic pain [4], yet it is still poorly recognised and treated. This reiterates the need and importance to develop an effective means to recognise and evaluate pain in this population [5]. Less accurate and valid self-report of pain in individuals with dementia has led researchers to develop instruments which help to recognise presence and severity of pain. Appropriate treatment and management of pain is a fundamental human right [6] and therefore not providing it is unethical. Persistent and untreated pain in dementia has been linked with an increased level of cognitive deterioration [7], which may lead to premature death.

In addition, up to 90 % of people with dementia express Behavioural and Psychological Symptoms of Dementia (BPSD), which include symptoms such as hallucinations, delusions, anxiety or depression and their presence has been associated with increase in psychiatric referral, incorrect use of antipsychotic medication and increased healthcare costs [8]. BPSD are commonly associated with cognitive decline in dementia, with symptoms usually being present from early stages of dementia and gradually worsening over time which is therefore negatively impacting life and the progress of the condition [9, 10]. Pain has a negative impact on BPSD, which in turn has a negative impact on aspects of daily living in people with dementia [11]. In such cases, effective pain management is needed to reduce BPSD such as depressive symptoms, anxiety, stress or agitation.

It should also be noted, that BPSD have been reported to be a major source of distress for caregivers and family members [12]. Informal family carers perceive BPSD as challenging as the symptoms are often associated with declining relationship between the person with dementia and the family members, suggesting an inevitable deterioration of the person’s condition [13].

Current observational pain assessment instruments are mostly paper-based and rely on observation and correct identification of presence and severity of pain indicators by the assessor. While an observational pain assessment is not the same as self-report of pain, observational pain assessment instruments can be useful to assess and monitor changes in severity of pain over time [14]. However, many of the instruments have methodological and practical shortcomings [15].

Several factors which could influence appropriate pain assessment and treatment have been identified [16] which included racial and ethnic disparities. While there are some underpinning physiological mechanisms involved, pain is largely a subjective experience, and as such, observer judgement is a factor which could hinder pain assessment due to observer’s previous knowledge, experience and bias of pain [17] suggesting that some observational pain assessment instruments are subjective, have limited evidence of accuracy, lack consistency, may be subject to bias and are underutilised in practice.

To increase the accuracy of pain assessment, these shortcomings and biases need to be addressed. PainChek® has been developed as a potential solution; it is a point-of-care application (App) which uses artificial intelligence and smart automation, to limit some of the factors which could hinder objective pain assessment [18]. PainChek® minimises human error and bias through the use of automated facial recognition and analysis to detect in real-time facial micro-expressions called action units (AUs), derived from the Facial Action Coding System [FACS] [20], which are indicative of pain. After detecting pain related facial AUs, the user then goes through a series of digital checklist and enters the presence of other non-facial pain cues covering the following domains: The Voice, The Movement, The Behaviour, The Activity, and The Body. To minimise inter-rater variability, definitions for each item are provided within the App and a binary scoring system is used (i.e. is this behaviour present: Yes = 1 and No = 0). The App collates the data from the six domains to automatically compute a total pain score. The overall pain score is then used to assign the severity of pain, where a score of 0–6 indicates no pain, 7–11 indicates mild pain, 12–15 indicates moderate pain and 16–42 indicates severe pain based on calibration against the Abbey Pain Scale (APS). More details on how the PainChek® tool works has been published elsewhere [19]. PainChek® has previously undergone psychometric [18, 20] and clinimetric [21] evaluations in Australia, and has demonstrated significant potential for use in the assessment of pain in people with moderate-to-severe dementia when compared to the APS. In 2018 the Royal College of Physicians, British Pain Society and British Geriatric Society provided guidelines for observational pain assessment in older people with dementia. While the guidelines did not provide a recommendation for a single observational pain assessment tool, it included a practical suggestion for the APS, among other tools within the UK [22].

In addition, the APS has been selected as a suitable clinical reference for this study for several reasons. Firstly, while there is no known single ‘gold standard’ for an observational pain assessment tool, recent UK guidelines recommend either the APS or Pain Assessment in Advanced Dementia Scale (PAINAD, 23). Additionally, the APS has been recognised as one of the better available observational pain assessment tools in terms of psychometric properties [5, 23–29]. The APS also includes at least one item from each of the six domains recommended by the American Geriatric Society [AGS] (see Table 1). Secondly, the APS is commonly used in the UK and other English speaking countries. Lastly, previous studies in Australia demonstrated the validity and reliability of PainChek® when compared to the APS [19, 20, 22, 30].

**Table 1:** Comparison of pain domains and items of American Geriatrics Society and PainChek®

Pain Behaviour Domains and Items	
AGS[19]	PainChek® [26]
**Facial expression** Slight frown; sad, frightened face Grimacing, wrinkled forehead, closed or tightened eyes Any distorted expression Rapid blinking	**The Face** (9 items based on FACS allowing automated analysis) Brow lowering (AU 4) Cheek raising (AU 6) Tightening of eyelids (AU7) Wrinkling of nose (AU 9) Raising upper lip (AU 10) Lulling of corner lip (AU 12) Horizontal mouth stretch (AU 20) Parting lips (AU 25) Closing eyes (AU 43)
**Vocalisation/Verbalisation** Sighing, moaning, groaning Grunting, chanting, calling out Noisy breathing Asking for help Verbally abusive	**The Voice (9 items)** Noisy sounds e.g. ouch, ah Requesting help repeatedly Groaning Moaning Crying Screaming Loud talk Howling Sighing.
**Body movements** Rigid, tense body posture, guarding Fidgeting Increased pacing, rocking Restricted movement Gait or mobility changes	**The Movement (7 items)** Altered or random leg/arm movement Restlessness Freezing Guarding/touching of body parts Moving away Abnormal sitting/standing/walking Pacing/wandering
**Changes in interpersonal interactions** Aggressive, combative, resisting care Decreased social interactions Socially inappropriate, disruptive Withdrawn	**The Behaviour (7 items)** Introvert (unsocial) or altered behaviour Verbally offensive Aggressive Fear or extreme dislike of touch, people Inappropriate behaviour Confused Distressed behaviours.
**Changes in activity patterns or routines** Refusing food, appetite change Increase in rest periods Sleep, rest pattern changes Sudden cessation of common routines Increased wandering	**The Activity (4 items)** Resisting care Prolonged resting Altered sleep Altered routine
**Mental status change** Crying or tears Increased confusion Irritability or distress	**The Body (6 items)** Profuse sweating Pale/flushed (red faced) Feverish/cold Rapid breathing Painful injuries Painful medical conditions

Taking into consideration the previous concerns around observational pain assessment instruments and the need for a highly valid means of assessing pain in people with advanced dementia, the rationale for this research was to further investigate the validity and reliability of PainChek® in a UK care home setting with a British cohort, using an Apple iOS operating system which had not previously been clinically validated. As such, the aim of this study was to further validate the psychometric properties of the PainChek® in UK a care home setting, using (Apple iOS version 2.14.1 (236) of the App), with a British cohort of individuals living with moderate-to-severe dementia.

## Methods

### Recruitment

Firstly, a nurse was recruited from the care home to assess pain using the APS during the data collection period. The nurse was selected on the basis of previous experience, skill, training, level of familiarity with residents and knowledge of dementia. The recruited nurse had been working in the care home for six years, was highly familiar with the structure, dynamics and residents within the care home and had completed in-house pain in dementia training. Furthermore, the nurse was highly familiar with the residents’ typical and atypical behaviours and was trained and experienced in using the APS.

Secondly, participants were recruited from a UK care home using opportunity sampling (see Table 2 for criteria). Due to the nature of dementia, the participants lacked capacity to give informed consent and were unable to comprehend the procedures of the study. Hence, informed consent was obtained through their personal consultee, legal guardian or Power of Attorney, which is in line with Mental Capacity Act guidelines [31]. The individual who provided informed consent on behalf of the participant was most often a close relative who came to visit the participant on a regular basis. Ethical approval was granted by the University of Derby College Research Ethics Committee, as well as the NHS Research Ethics Committee.

**Table 2:** Inclusion and exclusion criteria for this study

Inclusion criteria	Exclusion criteria
Diagnosis of moderate or severe dementia (measured by baseline MMSE score ≤ 20)	Individuals diagnosed with Parkinson’s Disease
Documented history of chronic pain condition (e.g. arthritis) OR residents which are often treated for pain due to painful complaints	Individuals partially or fully unable to exhibit facial features (such as some stroke survivors or those with facial deformities)
Residents of 65 years of age or older	Individuals with a significant mental health condition such as severe depression, anxiety disorders or schizophrenia which could result in unnecessary distress
	Individuals who have been advised not to take part by their GP, staff or family member

Individuals diagnosed with Parkinson’s Disease were excluded, as their facial features could have been compromised due to the nature and progress of the condition [32]. In addition, individuals with facial palsy, facial deformities or who were partially or fully unable to exhibit facial features were excluded. The inclusion and exclusion criteria were based on a previous study [30].

### PainChek®

PainChek® is a Class 1 medical device with a regulatory clearance in Australia (Therapeutic Goods Administration), Europe (CE Mark) Canada (Health Canada) and Singapore (Health Sciences Authority) for the assessment of pain in individuals who are unable to verbalise their pain, such as those living with moderate to severe dementia. It is an observational pain assessment tool which consists of 42 items spread across six domains; namely The Face (*n* = 9), The Voice (*n* = 9), The Movement (*n* = 7), The Behaviour (*n* = 7), The Activity (*n* = 4) and The Body (*n* = 6) [30]. Items included in PainChek® cover all six pain domains recommended by the AGS [21] for accurate and reliable observational pain assessment in people with cognitive impairment (See Table 1). However, the items in The Face domain have been based around the FACS, because as reported by Beach et al. [33], behavioural pain scales that have objective facial measures have better psychometric properties, than those containing vague facial descriptors.

PainChek® is simple to use. Users are provided with on device PainChek® User Guide and there is an online training module available at www.painchek.com. Users also undergo one and a half hours of training delivered either face-to-face or online which covers pain in dementia, what is PainChek® and how to complete a PainChek® assessment conducted by a PainChek® clinical consultant.

For this study, PainChek® (Apple iOS version 2.14.1 (236) of the App) was installed on Apple iPhone 6s (iOS version 12.2). This was the first clinical evaluation of the iOS version of PainChek® application undertaken in a different geographical location.

### Procedure

Prior to the recruitment process, the researcher spent four months visiting and observing processes and routines in the care home, this helped with familiarisation and integration of the researcher into the care home.

Once consent was given, baseline MMSE scores were collected. Paired pain assessments were collected by the two assessors independently, but at the same time for each participant at rest and immediately post-movement. The two assessors were blinded to each other’s assessment results. Paired ratings refer to a total of four pain assessments taken during one session; two assessments (one from APS one from PainChek®) were obtained during a restful state (e.g. participant sitting or lying down), followed by two assessments (one from APS one from PainChek®) obtained immediately after movement (e.g. participant was asked to stand up and sit down, or after a transfer from bed to a wheelchair). The paired assessments were collected from participants over a period of 16 weeks. Once all paired pain assessments were completed, final MMSE assessments for each participant were collected and debrief letters were sent out to legal guardians or those with Power of Attorney.

### Data Analysis

All statistics were analysed using IBM SPSS-26. The demographics of study participants and raw data were analysed using standard descriptive statistics, including mean, median, mode, range, standard deviation and frequencies or percentage for pain diagnoses and types of dementia.

Several validity and reliability measures were investigated. Firstly, concurrent validity was assessed by investigating Pearson’s correlation coefficients between the overall scores collected by APS and PainChek® as well as separately for the rest and post movement pain scores. This measure assesses the strength of a linear association between two variables. As the two instruments had different scoring systems the correlation is not an indication of a total agreement, but a strong and significant correlation would indicate that there’s a consistent increase or decrease of pain score in both instruments. Correlation coefficients were interpreted as follows: values 0-0.3 indicate negligible correlation, 0.3–0.5 low positive correlation, 0.5–0.7 moderately positive correlation, 0.7–0.9 high positive correlation and 0.9-1 very high positive correlation [34].

Secondly, interrater reliability was assessed. Cohen’s kappa [35] statistic was used to assess total agreement for the following categorical data: no pain, mild pain, moderate pain and severe pain. The Kappa scores were interpreted as follows: values ≤ 0 indicate no agreement, 0.01–0.20 indicate none to slight agreement, 0.21–0.41 as fair, 0.41–0.60 as moderate, 0.61–0.80 as substantial and 0.81–1.00 as almost perfect agreement [32].

Next, intraclass correlation coefficient (ICC) measured the reliability of ratings. The ICC ranges from 0 to 1, where a value closer to 1 indicates a higher similarity between values from the ratings of the same group. Values ≤ 0.5 are indicative of poor reliability, 0.50–0.74 indicate moderate reliability, 0.75–0.90 indicate good reliability and > 0.90 indicate excellent reliability [36].

Lastly, internal consistency was measured using Cronbach’s alpha (α), which examined whether the two instruments were measuring the same constructs. Cronbach’s alpha was computed based on overall scores of the two instruments, as well as rest and post-movement scores. Hulin et al. [37] suggest that α of 0.60–0.70 generally indicates an acceptable level of reliability and α ≥ 0.80 indicates a very good level of reliability.

In addition, discriminant validity was also investigated. This was achieved by looking at comparisons between pain scores at rest compared to post-movement for PainChek® and APS. A significant result (p ≤ .05) indicates that the observational pain instruments are activity dependent.

## Results

### Demographic characteristics of the participants

Overall, 302 paired assessments were collected from 22 participants. Of these, 179 assessments were conducted during rest and 123 were conducted immediately post-movement. The participants had a variety of dementia diagnoses, pain conditions and demographic characteristics as shown in Table 3. Given the demographic prevalence of dementia, it is unsurprising that the majority of the participants were female (77 %). The first MMSE assessment was obtained at baseline (Week 1), and final MMSE assessment was obtained at the end of data collection (Week 16). At the time of enrolment three residents were classified as having moderate dementia and 19 severe dementia based on their MMSE scores. During the course of the study, two residents died, both of whom had severe dementia (MMSE scores of 2 and 4, respectively).

**Table 3:** Clinical and demographic characteristics of participants

Characteristics	
Mean age (SD), years Median age (range), years	84.7 (5.6) 85.5 (74–95)
Sex, N (%) Male Female	5 (23) 17 (77)
Ethnicity, N (%) White British Black British	21 (95.5) 1 (4.5)
Baseline mean MMSE score, (SD), *N* = 22 Median MMSE (range)	5.78 (5.3) 4.5 (0–17)
End of study mean MMSE score, (SD), *N* = 20 Median MMSE (range)	3.6 (4.5) 2 (0–14)
Mean length of residency (SD), months at baseline Median lengths of residency (range)	25.8 (25.5) 14.5 (3–83)
Diagnosis of dementia, N (%) Alzheimer’s Disease Mixed Dementia Vascular Dementia Other/Unspecified Dementia Korsakoff’s Dementia	10 (45.5) 6 (27.3) 3 (13.6) 2 (9.1) 1 (4.5)
Diagnosis of pain conditions, N (%) Arthritis Osteoporosis Osteoarthritis Other musculoskeletal pain*	5 (22.7) 6 (27.3) 2 (9.1) 11 (50)

* Other musculoskeletal pain included lower back pain, tendonitis, stress fractures and bed sores

### Correlation between the APS and PainChek® for overall pain scores

The overall scores (i.e. pain assessments taken both during rest and immediately post-movement) were analysed. The overall mean for PainChek® pain scores were higher (M = 6.73, SD = 2.66) than the overall mean for APS pain scores (M = 3.57, SD = 1.42).

Pearson’s correlation coefficient revealed a significant correlation between overall PainChek® pain scores and overall APS pain scores (*r* = 0.82, *N* = 302, *p* < .001, one-tailed). In addition, the following reliability measures were tested; internal consistency (α = 0.81), single measure ICC (0.68; 95 % CI: 0.62 − 0.74) and inter-rater agreement (κ = 0.72).

### Correlation between the APS and PainChek® pain scores post-movement

In line with the direction of previous findings, the mean was higher for PainChek® post-movement scores (M = 7.91, SD = 2.58) compared to APS (M = 4.12, SD = 1.60).

Concurrent validity was tested using Pearson’s correlation coefficient, which demonstrated a significant relationship for post-movement scores between PainChek® and APS (*r* = 0.81, *N* = 123, *p* < .001, one-tailed). Internal consistency (α = 0.84), intraclass correlation for single measures (0.73; 95 % CI: 0.63 − 0.80) and an interrater agreement (κ = 0.84) were also tested.

### Correlation between the APS and PainChek® pain scores at rest

The means for resting assessment pain scores were higher for PainChek® (M = 5.91, SD = 2.40) than APS (M = 3.18, SD = 1.14). Pearson’s correlation coefficient for the rest condition also demonstrated a significant correlation between APS and PainChek® pain assessment scores at rest (*r* = 0.79, *N* = 179, *p* < .001, one-tailed). Interrater agreement (α = 0.76), single measures intraclass correlation (0.62; 95 % CI: 0.52 − 0.70), and agreement (κ = 0.64) were also tested.

In addition, in terms of discriminant validity, the difference in scores at rest and post-movement for PainChek® (*p* < .001) and APS (*p* < .001) were both significant.

## Discussion

This psychometric evaluation study of PainChek® provides further evidence for the suitability of PainChek® as a pain assessment instrument for individuals living with moderate-to-severe dementia. In addition to existing literature, this study provides evidence specifically related to the UK setting, in which the PainChek® has not previously been tested. Our findings provide further evidence that PainChek® has strong concurrent validity [34], high interrater agreement [35], good to very good internal consistency [38] and a moderate level of intraclass correlation [36]. Such findings support those from previous validation studies [18, 20, 30]. It is important to note that PainChek® consistently demonstrated these properties both when used to assess pain at rest as well as post-movement, findings which are consistent with previous validation studies [18, 20, 30]. This was also evidenced by the statistically significant elevation of pain scores in the post-movement condition compared to the rest condition for both tools. Further, pain scores immediately post movement were significantly higher than those undertaken at rest. This finding is also consistent with previous studies and provides evidence of the tool’s discriminant validity [18, 27]. Please note the care of residents and any decisions regarding their pain when they scored highly during observational pain assessment management was dependent on the result of the APS and the communication of the nurse with the rest of the team, not the researcher.

In comparison to the previous PainChek® studies [18, 30], overall in this study, the PainChek® has demonstrated a slightly lower concurrent validity. This could be partially explained by more closely examining the rest and the post-movement conditions. This study, like many others, has further demonstrated that pain behaviours are best elicited by movement [39, 40], hence the consistently lower scores in the rest condition could be explained by the difficulty of obtaining an accurate pain score during a resting condition.

In terms of internal consistency, the results from the present study demonstrated slightly lower agreement at rest, but higher agreement in the post-movement condition when compared to Atee et al. [27]. Other observational assessment tools which have demonstrated similar interrater agreement are the NOPPAIN [41], MOBID [42] and previous PainChek® studies [30].

Overall interrater agreement scores were lower during the rest condition than the post-movement condition. This is consistent with previous literature indicating that pain is easier to detect once elicited by movement [39, 40]. In addition, the pain assessment tool PAINE [43] had a similar interrater agreement scores to those found within this study.

The comparison of the baseline and end of study MMSE scores demonstrated a deterioration in cognition of all participants over the 16 weeks period of the study. This is in line with expected deterioration of cognition as a result of the progression of dementia [44]. This finding further evidences the reliability of PainChek®, as it continued to consistently identify the presence and severity of pain over the 16-week data collection period, despite the progression of dementia and the associated deterioration of cognition. There are clinical implications for this finding, suggesting if PainChek® is used on regular basis in UK care homes, it is able to continuously provide an accurate pain assessment for individuals over time, regardless of deterioration of symptoms or functional status, therefore overcoming the potential human error and bias which may occur with healthcare professionals who have daily contact with the individuals. Overall, this could facilitate pain management through improved pain assessment and better use of pain related pharmacological and non-pharmacological interventions.

In terms of concurrent validity, previous researchers [5] specified adequate observational pain assessment instruments should demonstrate at least a score of 0.4–0.6, which PainChek® has exceeded. Observational pain assessment instruments, such as the MOBID-2 [45] or the Pain Assessment Checklist for Seniors with Limited Ability to Communicate (PACSLAC) [46] and many others as outlined in a systematic review [25] have also demonstrated concurrent validity values which meet the specified adequate range as outlined by Herr et al. [5]. However, research demonstrated inconsistencies between observational pain assessment instruments, specifically in the facial expression domain [46, 47], due to their subjectivity. It has also been suggested that better accuracy and precision would benefit observational pain assessment instruments and that this could be achieved by further refining and developing the facial expression domain [47]. PainChek® has an automated facial expression decoding function, therefore mitigating the chance of non-agreement between raters by utilising facial recognition technology, which does not rely on manual input of pain presence and severity. This gives PainChek® an advantage over other observational pain assessment instruments which require the assessor to score the facial expression manually, as the face domain is often problematic, difficult to score and remains to be one of the most poorly scored domains carrying the highest level of subjectivity, in comparison to other pain domains in observational pain assessment instruments [48].

PainChek® is considered to be a screening tool, which acts as a guide for users as part of future decision-making process relating to pain management and treatment of residents with dementia. The instrument informs the user about the intensity of present pain, which adds to its clinical utility in that the choice of analgesics is often based on the intensity of the pain detected. The cut-off points for different pain intensities have previously established against the APS [21]. However, to date no studies have been reported that evaluated the impact of PainChek® use on clinical outcomes, although this is planned as part of a major implementation trial in aged care currently underway in Australia.

In terms of study limitations, the study aimed to be pragmatic in terms of being as realistic and practical as possible, to help replicate everyday care home dynamics. For example, participants were not taken into a separate quiet room during pain assessments, as this would have been unrealistic and unsustainable for this particular care home. When residents show behaviour, which could be associated with pain, the initial assessments are conducted in the communal areas of the care home. Because of this, the administration of PainChek® and APS were also completed in the communal areas, to replicate a realistic approach to pain assessment in care homes. Some generalisability limitations are present as it is a single site study involving one care home. Although participants with a variety of dementia diagnoses, levels of severity and a range pain diagnoses and conditions were recruited, all assessment were completed by the same two raters. A larger study involving multiple trained nurses across multiple care homes should be conducted to increase the overall generalisability and ecological validity of the findings.

In addition, multiple attempts have been made in the design of PainChek® to minimise potential human error. This includes use of automated facial analysis, binary scoring, in app definitions of each item, forcing users to complete each domain and automated computing of pain scores and assignment of pain intensities. Additionally, users are also provided with structured training to ensure their confidence and competence in completing PainChek® assessments.

Strengths of this study include the observation visits prior to data collection, which took place one to three times a week over a period of four months. This enabled the researcher to become familiar with the environment, procedures, dynamics, staff and residents, but also helped to understand how PainChek® could be implemented within the care home’s daily processes and protocols in the future. The observational period took place from September 2018 until January 2019, during this time the researcher was often involved in small activities with the residents, such as preparing tea and coffee, helping to feed residents during mealtime and talking with residents who were verbal. This enabled the researcher to collect highly ecological and organic data, as the participants reacted to the researcher with the same familiarity as they would have reacted to a staff member.

## Conclusions

In conclusion, this study provides evidence of PainChek®’s psychometric properties when used within the UK home care setting, therefore supporting its previously reported validity and reliability from studies conducted in Australian residential aged facilities. This instrument has the capacity to empower caregivers to accurately assess, treat and manage pain in care homes. As such, PainChek® has the potential to support healthcare providers to improve quality of life in the population with dementia.

## Data Availability

The PainChek® datasets used and analysed as part of this study are not publicly available.

## Abbreviations

APS: Abbey Pain Scale
GP: General Practitioner
ICC: Intraclass Correlation Coefficient
iOS: iPhone Operating System
MMSE: Mini-Mental State Examination
NHS: National Health Service
PACSLAC: Pain Assessment Checklist for Seniors with Limited Ability to Communicate
